# Risk factors for unintentional injuries among the rural elderly: a county-based cross-sectional survey

**DOI:** 10.1038/s41598-017-12991-3

**Published:** 2017-10-02

**Authors:** Hongping Zhang, Feng Wei, Mo Han, Jianquan Chen, Songxu Peng, Yukai Du

**Affiliations:** 1 Department of Social Medicine and Health Management, School of Public Health, Tongji Medical College, Huazhong University of Science & Technology, HangKong Road 13, Wuhan, 430030 China; 2 Centers for Disease Prevention & Control of Huangpi District of Wuhan, BaiXiu Street 255#, Huangpi District, Wuhan, 430300 China; 3 Department of Disease Control, Health and Family Planning Commission of Huangpi District of Wuhan, BaiJing Street 224#, Huangpi District, Wuhan, 430300 China

## Abstract

This study aimed to provide evidence for the prevention and reduction of unintentional injuries in the rural elderly by analysing epidemiological data of injuries among rural older adults (65^+^) and identifying the involved risk and protective factors. This study analysed all information, including the social demographic characteristics, chronic disease condition, lifestyle, living environment, mental health, activities of daily living and detailed information about the nature of the injuries. Chi-square tests, rank tests and a multivariate logistic regression were performed. The prevalence of unintentional injuries was 44.4%; according to the multivariate regression analysis, ten variables, including gender, floor tiles, cane use, sleeping duration, roughage intake frequency, mental health status, diabetes, arthritis and cataracts, were involved in the injury patterns. Low roughage intake (OR = 2.34, 95% CI 1.64–3.35), the use of a cane (OR = 1.78, 95% CI 1.31–2.41), a sleeping duration of five hours (OR = 1.75, 95% CI 1.27–2.42) and severe mental disorders (OR = 1.61, 95% CI 1.01–2.57) were the top 4 risk factors. In conclusion, we found that unintentional injuries among the rural elderly were closely related to chronic disease, mental health and residence environment. These findings could be beneficial for the prevention of unintentional injuries and for policy makers and health service managers.

## Introduction

Unintentional injuries among older adults are an increasing public health concern due to the overall ageing of the population. The mortality rate due to injury has increased over the past decade among adults aged 65 years or older^1–4^. According to Karb, the mortality rate (per 100,000) due to all unintentional injuries among adults aged 65–84 years was 66.87, which is approximately 10 times that observed among children aged 0–14 years; moreover, the mortality rate among those aged 85 years or older was 337.27, which is nearly 50 times that observed among children aged 0–14 years in the United States between 1999 and 2012^5^. In Korea, injury-related mortality has increased among adults aged 65 years or older. In particular, injury-related mortality among women older than 80 years has doubled since 1996. Falls replaced transport as the leading cause of injury-related deaths among the elderly^6^. In addition, the elderly are often afflicted with a variety of diseases, such as cardiovascular disease and diabetes, that reduce the likelihood of survival in the case of non-fatal injuries. Furthermore, the increasing rates of emergency department (ED)-treated and the high number and proportion of inpatient trauma days due to unintentional injuries among elderly adults poses a challenge to the health care system and increases the economic burden on society^3,7,8^. Therefore, more effective measures are needed to prevent and reduce unintentional injuries among elderly people and minimize the negative health effects and increasing health costs.

Age has been considered a risk factor for unintentional injuries among the elderly because the risk of unintentional injury is higher among the older-elderly population^6,9–11^. However, age has also been reported to have no influence on the rate of injuries among the elderly^3,12^. According to Saveman, the injury rate among elderly females is higher than that among elderly males^10^; the unintentional fatal injury rate is higher among females than that among males; and females older than 70 years are more likely to fall than males, leading to a greater likelihood of bone fractures^2^. Certain chronic diseases, including lumbar spondylosis, orthostatic hypotension, diabetes and cataracts, are risk factors for falls among elderly individuals^13^. Severe bilateral visual deficits increase the risk of unintentional mortality among adults over the age of 18 years (including the elderly)^14^. Depression^15–17^ and other mental illnesses^18^ are related to the risk of injuries. Elderly individuals with Alzheimer’s disease are more prone to unintentional poisoning^19^. The use of medications also explains the increased rate of unintentional injuries among the elderly; for example, elderly individuals who consume opioid drugs are more likely to fall^20^. Furthermore, sleep duration affects unintentional injuries among adults; sleeping less than 6 hours was a risk factor for unintentional injuries^21^. The fall risk among elderly individuals is closely related to the activities of daily living (ADL) capability, physical activity habits, poor living conditions and environmental factors^22^. Furthermore, poverty at the county level confers a greater risk of unintentional injury, and high poverty areas have shouldered the burden of the recent national increases in the mortality rate due to unintentional injuries^2,5,23^. In India, more than 80% of unintentional injury-related deaths occurred in rural areas^2^. There is an increasing socioeconomic disparity among all combined unintentional injuries. The injuries observed in rural areas and among individual from low socioeconomic classes are more severe^24^.

In China, most elderly people continue to live in the countryside. Because the young adult labour forces have migrated from the rural areas to the cities, the elderly population remaining in the rural areas must live alone without the support of their adult offspring (usually referred to as “empty nesters”). More attention should be paid to the older rural population because this population is more likely to experience unintentional injuries. Most elderly people work as active farmers under poor economic conditions with less support from younger adults. Population-based surveys covering the range of injuries among older rural adults are scarce^10^. Our study attempts to address these shortcomings.

In this study, we performed cluster sampling using our self-designed questionnaire. The questionnaire assesses social demographic characteristics, financial situations, conditions of offspring, prevalence of chronic disease, living environment, mental condition, ADL and instrumental ADL (IADL) among elderly individuals. This study aims to identify policies, programmes, and resources that ensure a safe environment and promote safe behaviours to prevent injury. Therefore, the situations responsible for unintentional injuries and the risk and protective factors that minimize these injuries were analysed. Comparisons of specific injury-related mortality between states should be conducted with caution because the large differences in the unspecified injury mortality rates across states could create a bias^4^. Furthermore, unintentional injuries are not homogeneous phenomena from an epidemiological transition perspective^25^. This study was conducted amongst the rural population in one entire county under the jurisdiction of Wuhan City, Hubei Province, China.

## Results

### General description of the investigation

Of the 3,900 questionnaires distributed, we received 3,752 completed questionnaires, representing a response rate of 96.2%. The database included 1,673 males (44.6%) and 2,079 females (55.4%), and the average age of these individuals was 72.74 ± 6.44 years, with a range of 65 to 100 years. Of those surveyed, 64.8% were 65–74 years old, and 5.3% were 85 years old or older. In total, 1,665 injuries were reported by 805 victims in a sample of 3,752 respondents aged 65 years and older during the 12-month period covered by this investigation. The observed injury prevalence in this rural elderly population was 44.4% (Table 1). The questionnaire responses included 487 cases (59.8%) of one injury, 127 cases (15.8%) of two injuries and 191 cases (23.7%) of three or more injuries. Falls (30.0%) and cuts (5.4%) were the most common unintentional injuries, followed by choking or swallowing a foreign body (4.4%), burns (1.0%), traffic accidents (0.8%), and sunstroke (0.8%) (Table 1).

**Table 1:** Prevalence of different types of unintentional injuries by age and gender among the study population.

Causes of injuries	Total	Sex			Age group in years (y)			
	N = 3752 n (%)	Male N = 1673 n (%)	Female N = 2079 n (%)	*P*-value	65–74 N = 2431 n (%)	75–84 N = 1122 n (%)	≥85 N = 199 n (%)	*P*-value
Total	1665(44.4)	578(34.5)	1087(52.3)	0.000**	1035(42.6)	548(48.8)	82(41.2)	0.130
Falls	1124(30.0)	347(20.7)	777(37.4)	0.000**	639(26.3)	430(38.3)	55(27.6)	0.000**
Cuts	201(5.4)	79(4.7)	122(5.9)	0.476	149(6.1)	49(4.4)	3(1.5)	0.021*
Choking or swallowing a foreign body	164(4.4)	77(4.6)	87(4.2)	0.415	121(5.0)	33(2.9)	10(5.0)	0.195
Burns	36(1.0)	5(0.3)	31(1.5)	0.013*	23(0.9)	7(0.6)	6(3.0)	0.258
Traffic accidents	31(0.8)	20(1.2)	11(0.5)	0.004*	23(0.9)	7(0.6)	1(0.5)	0.485
Sunstroke	30(0.8)	12(0.7)	18(0.9)	0.435	23(0.8)	6(0.5)	1(0.5)	0.729
Crushing accidents	22(0.6)	13(0.8)	9(0.4)	0.044*	16(0.7)	6(0.5)	0(0.0)	0.630
Animal bites	19(0.5)	10(0.6)	9(0.4)	0.219	11(0.4)	7(0.6)	1(0.5)	0.706
Percussions	17(0.5)	3(0.2)	14(0.7)	0.326	11(0.4)	1(0.1)	5(2.5)	0.180
Poisoning	6(0.2)	5(0.3)	1(0.0)	0.055	6(0.2)	0(0.0)	0(0.0)	0.284
Drowning	4(0.1)	2(0.1)	2(0.1)	0.638	4(0.2)	0(0.0)	0(0.0)	0.367
Domestic violence	3(0.1)	0(0.0)	3(0.1)	0.262	1(0.0)	2(0.2)	0(0.0)	0.812
Electric shock	1(0.0)	1(0.1)	0(0.0)	0.206	1(0.0)	0(0.0)	0(0.0)	0.779
Other injuries	7(0.2)	4(0.2)	3(0.1)	0.504	7(0.3)	0(0.0)	0(0.0)	

^*^ *P* < 0.05; ^**^ *P* < 0.01; % = prevalence.

### Sex- and age-based injury patterns

The incidence of the unintentional injuries was significantly higher among the female respondents (52.3%) than among the male respondents (34.5%) (P < 0.001). Females were significantly more likely to experience fall-related injuries (37.4% vs. 20.7%, P < 0.001) and burn injuries (1.5% vs. 0.3%, P = 0.049). Males were significantly more likely to experience a traffic injury (1.2% vs. 0.5%, P = 0.004) and crushing injuries (0.8% vs. 0.4%, P = 0.044) than females (Table 1).

The prevalence of the injuries varied according to the age group and type of injury (Table 1). The group of participants aged 75–84 years had the highest rates of all injuries (48.8%), followed by those aged 65–74 years (42.6%), and the group of participants aged 85 years or older had the lowest rate (41.2%). However, no significant differences were observed among these groups (P = 0.130). The group aged 75–84 years had higher rates of fall-related injuries (38.3%) than the older group (27.6%) or younger group (26.3%) (P < 0.001). The group aged 65–74 years had higher rates of cut injuries (6.1%) than the older group (4.4%) and the oldest group (1.5%) (P = 0.010).

### Treatments and costs

Upper and lower limb injuries were the most commonly reported injuries, followed by head and neck injuries and spine-backbone/back injuries. Bruises, fractures and sprains were the top 3 most common injuries. The most common settings in which the injuries occurred were at home (40.6%) and the workplace (22.3%) (Table 2). In total, 44.4% (n = 357) of the study population sought medical help, 28.2% (n = 227) of the study population did not receive any treatment and 27.4% (n = 221) of the study population self-medicated. The total medical costs of the 1,665 injuries were 1,656,845 RMB; 144 people (20.3%) were admitted for 2,346 days in hospitals, representing an expenditure of 1,330,600 RMB. In total, 51.3% of the elderly was covered by the New Rural Cooperative Medical Care Scheme, 40.3% of the elderly paid for their treatments and 8.4% of the elderly used another health insurance carrier, such as free medical service or commercial insurance (Table 3).

**Table 2:** Comparison of the anatomical site, pathological type and location of the unintentional injuries between the genders in a rural elderly population.

`	Total		Sex				*P*-value
			Male		Female		
	N	%	N	%	N	%	
Total	3752		1673	44.6	2079	55.4	
Injuries							<0.001**
Yes	805	21.5	301	18.0	504	24.2	
No	2947	88.5	1372	82.0	1575	75.8	
Age (y)							
65–74	2431	64.8	1054	63	1377	66.2	0.026*
75–84	1122	29.9	537	32.1	585	28.2	
≥85	199	5.3	82	4.9	117	5.6	
Anatomical site of injury							
Head and neck	89	11	36	12.1	52	10.3	>0.05
Spinal bone and back	52	6.5	21	6.9	32	6.3	
Upper extremities	289	35.9	97	32.3	192	38	
Lower extremities	297	36.9	118	39.1	180	35.7	
Buttock	45	5.6	17	5.6	28	5.6	
Other anatomical sites	33	4.1	12	4	21	4.1	
Pathological type of injury							
Bruise	401	49.8	155	51.6	245	48.7	>0.05
Fracture	152	18.9	48	16.1	103	20.5	
Twist	147	18.3	51	16.9	96	19.1	
Open (penetrating trauma)	43	5.4	20	6.7	24	4.7	
Brain dysfunction	22	2.7	9	3.1	12	2.4	
Other	39	4.9	17	5.6	23	4.6	
Injury place							
At home	327	40.6	99	32.9	229	45.5	0.006**
Workplace (farmland)	180	22.3	80	26.7	98	19.5	
On the road	159	19.8	71	23.6	88	17.4	
In the yard	101	12.5	35	11.6	66	13.1	
Public place	19	2.4	6	2.1	13	2.6	
Other places	19	2.4	9	3.1	10	1.9	
Activity							
Recreational activity	350	43.5	99	32.9	255	50.6	<0.001**
Working	175	21.8	83	27.6	92	18.3	
Walking	77	9.6	36	11.9	44	8.7	
Exercising	43	5.3	17	5.7	25	5	
Sleeping/resting	39	4.9	16	5.4	23	4.6	
Cooking	55	6.8	20	6.8	34	6.7	
Riding	22	2.7	11	3.6	6	1.1	
Driving	15	1.9	10	3.2	6	1.1	
Other	28	3.5	9	2.9	20	3.9	

^*^ *P* < 0.05; ^**^ *P* < 0.01; % = rate.

**Table 3:** Comparison of the treatments and outcomes of unintentional injuries between the genders in a rural elderly population.

	Total		Sex				
			Male		Female		*P*-value
	N	%	N	%	N	%	
Treatment							
no treatment	227	28.2	85	28.1	143	28.3	>0.05
self-treatment	221	27.4	78	25.9	143	28.3	
town hospitals	127	15.8	49	16.4	78	15.4	
district hospitals	104	12.9	41	13.5	64	12.6	
village clinics	74	9.2	25	8.4	48	9.6	
city level hospitals or above	29	3.6	13	4.4	16	3.1	
private clinic	23	2.9	10	3.3	14	2.7	
Injury severity							
mild	581	72.2	222	73.6	359	71.3	>0.05
moderate	205	25.5	71	23.6	135	26.7	
severe	19	2.3	8	2.8	10	2	
Hospitalization							
yes	163	20.3	76	25.1	87	17.3	0.012
no	642	79.7	225	74.9	417	82.7	
Types of payments							
new type of rural cooperative medical system	413	51.3	157	52	257	50.9	>0.05
self-paying	324	40.3	117	39	207	41.1	
urban residents’ health insurance	51	6.3	15	5.1	35	7	
free medical service	12	1.5	8	2.8	4	0.7	
commercial insurance	5	0.6	3	1.1	2	0.3	
Limited function							
without limitations	336	41.8	139	46.1	197	39.1	>0.05
1–14 d	68	8.5	29	9.7	39	7.7	
15d-30 d	159	19.8	54	17.8	106	21	
1–3 m	49	6.1	16	5.3	33	6.6	
>3 m	122	15.2	43	14.2	80	15.9	

^*^ *P* < 0.05; ^**^ *P* < 0.01; % = rate.

### Risk factors

Our study identified 27 factors related to injuries by performing a single-factor logistic regression analysis with the occurrence of injuries as the dependent variable. This study subsequently assessed the above-mentioned 27 factors by performing a multivariable logistic regression analysis. According to this analysis, ten variables, including gender, the presence or absence of floor tiles, a residence near a road, use of a cane or walking stick, sleep duration, food intake, mental health, diabetes, arthritis and cataracts, were all factors in the injury patterns (presented in Table 4).

**Table 4:** Multivariate logistic regression analysis results: risk factors for injury.

	All Injuries		
	Odds ratio	95% CI	*P*-value
Female	1.46	1.20–1.77	0.000
Floor			
non-slippery floor	1.00		Reference
slippery floor	1.07	0.82–1.39	0.630
without floor	1.60	1.30–1.99	0.000
Domicile near road	1.37	1.13–1.66	0.001
Using a cane	1.78	1.31–2.41	0.000
Sleeping time			
≤4 h	0.88	0.60–1.30	0.526
5 h	1.75	1.27–2.42	0.001
6 h	1.45	1.06–1.98	0.021
7 h	1.01	0.72–1.41	0.952
8 h	1.10	0.80–1.51	0.557
≥ 9 h	1.00		Reference
Roughage intake frequency			
daily	1.00		Reference
often	1.70	1.18–2.43	0.004
occasionally	1.84	1.29–2.63	0.001
hardly	2.34	1.64–3.35	0.000
Mental capacity			
likely well	1.00		Reference
mild mental disorder	1.15	0.89–1.50	0.288
moderate mental disorder	1.59	1.09–2.32	0.017
severe mental disorder	1.61	1.01–2.57	0.045
Diabetes	1.42	1.08–1.88	0.013
Arthritis	1.27	1.02–1.59	0.032
Cataracts	1.38	1.06–1.79	0.017

In general, women had a 46% greater chance of injury than men, and these higher odds reached 72% for fall-related injuries. Compared with people with a non-slippery floor, elderly adults with slippery floors had a 10% higher probability of injury, and those who had no floor (i.e., those living in a dwelling with an earth or cement floor) had a 60% higher chance of sustaining an injury. Using approximately 9 hours of sleep as a reference, the adjusted injury risk (odds ratio) for those who sleep less than 4 hours/day was 0.88. The odds ratio was 1.75 for those sleeping 5 hours per day, 1.45 for those sleeping 6 hours per day, 1.01 for those sleeping 8 hours per day and 1.10 for those sleeping 9 hours or more per day. The risk difference was significantly different between the 5- and 6-hour cohorts. In this study, the risk increased as sleep duration decreased from 7 hours to less than 4 hours. The use of a cane or walking stick increased the odds of injury by 78%. The group with the lowest food intake had a 2.34-fold higher probability of sustaining an injury than the group with normal food intake. Individuals with severe mental disorders were 61% more likely to sustain recurrent injuries and 59% more likely to sustain an injury than the individuals in the moderate mental disorder group. People who had a chronic disease had a higher risk of injury as follows: individuals with diabetes had a 42% higher probability of sustaining an injury, individuals with arthritis had a 27% higher probability, and individuals with cataracts had a 38% higher probability.

## Discussion

This is the first study to investigate the rate of unintentional injury among rural elderly individuals using comprehensive information regarding the socio-demographic characteristics, chronic disease condition, lifestyle, living environment, mental condition, ADL and IADL. The prevalence of unintentional injuries was 21.5% in this rural elderly population. The top six unintentional injuries were falls, cuts, choking or swallowing a foreign body, burns, traffic accidents and sunstroke. Prior domestic and foreign studies mostly investigated the elderly in a city or community environment, in which falling and traffic injuries are the most common injury types^2,26^. By contrast, this study was conducted among the elderly in rural areas, and injuries from cuts ranked the highest because this population typically uses blades or other sharp-edged implements for agricultural production. Some elderly individuals use wood instead of gas or coal as fuel to heat their homes and cook. Therefore, these individuals are prone to injuries from blades as they prepare wood for burning. Because these individuals use wood for heat and cooking, they are also more prone to burns sustained when feeding the stove. Due to their age, their potential to experience a burn- or fire-related injury increases as a function of the ageing process, co-morbidities, and limited financial means^27^. These individuals are also prone to sunstroke while working outside. In this investigation, the incidence of swallowing a foreign body and choking was 1.6%, ranking as the third most common injury, which has not been observed in other studies. The elderly are more prone to dysphasia-choking than children, who are more prone to choke by swallowing a foreign body. Among unintentional deaths in Japan, choking ranks as the number one cause^28^. Among the approximately 800 choking cases attributed to food, mochi ranks the highest at 20%, and this rate is particularly high among elderly individuals aged more than 65 years. Mochi is a traditional Japanese food and an important festive feature during the New Year’s holiday, particularly among the elderly. Mochi is made by steaming sticky rice. Mochi is highly cohesive and adhesive and can easily lead to choking. The people in this Chinese study area also have a custom of eating a similar traditional food named “CiBa”, which is made by steaming sticky rice. The role of food in choking injury requires further investigation.

In the comparison of the injury types between the males and females, the females were more prone to experience falls (P < 0.001) and burn injuries (P = 0.013), and the males were more prone to experience traffic accidents (P = 0.038) and crushing injuries (P < 0.001). The most common settings in which the injuries occurred for both genders were at home and the workplace, but the incidence of females sustaining unintentional injuries at home was higher (P = 0.006). No differences were observed in the types of injuries and the location and severity of the injuries between the genders (P > 0.05). The males had higher rates of admission to health facilities than the females, implying either that the males paid more attention to their health status or that the females bore the pain more readily or lacked the authority to make financial decisions.

Ten variables were analysed to determine the injury patterns using a multivariate logistic regression analysis. These variables included gender, the presence or absence of floor tiles, a residence near a road, the use of a cane, hours of sleep, intake of food, mental health, diabetes, arthritis and cataracts (Table 4). The elderly women were more likely to suffer unintentional injuries than the elderly men, which is consistent with other studies^10,29^. Elderly individuals who used canes were more likely to sustain unintentional injuries, likely because those using walking sticks cannot walk freely and, thus, are more likely to be injured in falls or traffic accidents. Moreover, at least one arm is occupied using the walking stick, making it easier to experience burns or cuts. In this study, those with 5 hours or less of sleep had 1.85-fold higher odds of injuries, and those with 6 hours of sleep had 1.45 higher odds more chance of injuries than those with approximately 9 hours of sleep (P < 0.05). Similar results were obtained by Kim^21^ in his study, which investigated the relationship between hours of sleep and the likelihood of unintentional injuries. Similarly, sleep deprivation has been shown to lead to fatigue, drowsiness while driving, and impaired alertness, directly leading to an increased number of traffic accidents. Moreover, a decrease or increase in sleep duration has been shown to have an effect on health, and sleep duration is associated with reduced cognitive function^30,31^_._

Depression is related to the risk of injuries^15–17^, but other mental illnesses are also risk factors for unintentional injury and injury recidivism^18^. In this study, we used the Kessler 10-item Psychological Distress Scale (K10) to measure the level of mental functioning and evaluate the risk of mental problems^16,32,33^ in the study population because this test is applicable on a wider scale and has been validated in Chinese elderly populations in many studies^28,31,32^. This study used the K10 to determine the relationship between mental illness and the likelihood of injuries. The rate of injuries was higher in those with mental problems. Elderly Chinese men and women, particularly those from rural areas, are not prone to discuss mental disorders. The K10 includes only ten items; therefore, this test is easy to use in screening for mental disorders. The results of other studies^28,31,32^ have shown that this test is applicable to a wide range of mental disorders.

The location of a person’s residence in relation to a road is a contributing factor for the likelihood of injuries. Those located far from a road are more likely to be injured than those located close to a road. Those located far from a road experience more difficulties in accessing the more difficult terrain to cultivate mechanically. Therefore, a greater extent of manual labour is observed, which increases the likelihood of cuts and burn injuries.

The elderly individuals with uneven mud or concrete floors in their residences were more likely to suffer injuries than those with finished floors with anti-slip floor tiles. The absence of a finished floor in the house is a consequence of a lower economic status. The economic status of a household was not a contributing factor in the rural elderly likely because these individuals are reluctant to disclose their true economic situation.

The home is normally a place of safety. However, in this study, 45.5% of the elderly women and 32.9% of the elderly men sustained injuries at home. Certain risk factors are associated with the physical condition of the residence. Improving the economic condition and living environment and reducing the physical conditions that lead to an increased risk of injuries in the residence can effectively decrease the incidence of injuries.

Of the 17 types of common chronic diseases, the following 3 diseases were prominent in our injury and fall model: diabetes, arthritis and cataracts. Elderly individuals with these conditions require more care and are more prone to suffer unintentional injuries.

This study shows that daily roughage intake is a protective factor against unintentional injuries. No previous study has reported a relationship between the risk of injuries and regular roughage intake. Further studies must be performed to explore this relationship. Particular attention must be paid to the age of the elderly people studied. A growing body of research is examining ageing as a risk factor for falls or other unintentional injuries^7,10,34^. However, these studies indicate that ageing alone is not a risk factor for unintentional injuries. Age must be considered in the context of the functionality of an individual. Rural elderly individuals usually work outdoors and, thus, are likely to retain better ADL functions with age.

In summary, this study analysed the unintentional injuries sustained by elderly people in rural areas and found that unintentional injuries are closely related to chronic disease, mental health status and overall residential environment. The findings of this study could be beneficial for the prevention of unintentional injuries and hence could be vital for policy makers, health service managers and stakeholders.

## Methods

### Study design and setting

This study used a county-based, cross-sectional survey design, with a convenience sample collected from one county. We aimed to study the epidemiology of unintentional injuries among rural older adults (65 years or older) and analyse the risk and protective factors to provide evidence that can be used to prevent and reduce unintentional injuries and minimize the increasing health costs. The questionnaire was administered to a randomized, stratified sample in different stages and was proportional in age and population. A minimum sample size of 3,630 rural individuals aged 65 years and older is recommended. The sample size formula was N = Z _a/2_^2^ × π (1−π)/δ^2^. The sample size was obtained to achieve a confidence level of 95%, with a 5% margin of error and 50% prevalence of unintentional elderly injuries (no national studies have investigated the same age categories) with an expected response rate of 90%. We considered mountainous areas, flatlands and economic level as the layering factors.

According to the different geographical locations (flatlands and mountainous areas) and economic level (good, medium and poor) (data on the per capita income of the rural residents was obtained from the *Huangpi district statistical yearbook* (2015)), 2 townships were selected from each level, and 2 villages were randomly selected from each township. Finally, all qualified elderly people from 24 villages were selected as the study population. We arranged for those aged between 65–79 years to be surveyed together in health clinics in their towns, and in-home surveys and other appropriate methods were used to survey those over 80 years of age or those who were physically inconvenienced. Huangpi County is a county in Wuhan City in Central China, and the net per capita income of the local farmers is in the middle level of that in Hubei Province. Generally, this region is composed of “half mountain, half field”, indicating that mountains and plains exist in the same half area. Therefore, Huangpi County has a certain rural representation of rural county in the terrains and landforms.

In this study, we defined unintentional injuries as falls, traffic accidents, cuts, burns, swallowing a foreign object or choking, sunstroke, animal bites, electric shocks, crushing injuries, getting hit by an object, poisoning, drowning, domestic violence and other injuries. The expected timeframe for completing the survey was one year, and the study was performed between September 1^st^, 2015 and September 1^st^, 2016.

### Data collection procedures

Sixteen medical postgraduate students were recruited and trained as investigators and interviewers to collect the relevant information. The participants were predominantly elderly individuals (95.6%). If the elderly person could not communicate because of disease or language or hearing problems, the immediate relatives or another responsible adult member of the household completed the interview. The participants were asked about any history of injuries during the last 12 months. For inclusion in the study, the injury was determined according to one of the following circumstances: (a) if the elderly person was injured and treated with simple medical therapies by themselves or another relative; (b) if the injury was diagnosed by a doctor or nurse in a clinical setting; or (c) if the individual rested or remained in bed for a minimum period of 4 hours or a half a day because of the injury. To ensure the quality of the collected data, this study was first conducted as a small-scale pilot study, which involved 150 samples from one village after designing the questionnaire. This pilot study was also used to train the research staff in managing the data collection and data entry. After this pre-test, the questionnaire was modified.

### Questionnaire

The questionnaire consisted of the following 5 parts: Part A covered the personal social demographic characteristics, including age, gender, nationality, marital status, education, occupation, financial situation and offspring condition, of the elderly adults. Part B included information regarding daily habits and chronic disease conditions. Part C collected detailed information regarding the nature of the injuries, the living environment, the injury treatment and associated cost. Part D used the K10 to examine the mental condition of the elderly adults. The K10 is a questionnaire designed to identify individuals likely to have a diagnosed or undiagnosed mental illness with symptoms that are severe enough to cause moderate to severe functional impairment. The K10 is embedded in the Sample Adult Questionnaire. Each of the ten components has the same possible responses including 0 (“none of the time”), 1 (“a little of the time”), 2 (“some of the time”), 3 (“most of the time”), and 4 (“all of the time”). The scores are summed to obtain a score between 0 and 40. In our study, the psychological distress level was categorized as follows: 0–5 points indicate that the individual is functioning at a normal level, 6–11 points indicate mild mental distress, 12–19 points indicate moderate mental distress, and 20–40 points indicate severe mental distress. Part E included an ADL scale and an IADL scale. Independence in ADL was identified using the Barthel Index (BI). The BI is a questionnaire consisting of 10 items that include feeding, bathing, grooming, dressing, continence of bowels and bladder, transferring from wheelchair to bed and return, transferring to and from a toilet, walking on a level surface, and going up and down stairs. The total score of the BI ranges from 0–100, with a score of 0 indicating completely incapable of ADL, a score of 1–40 indicating seriously dependent ADL, a score of 41–60 indicating moderately dependent ADL, and a score of 61–100 indicating the individual is capable of independent ADL. The Lawton IADL a questionnaire assesses the following 7 IADL functions: using the telephone, using a transportation method, managing money, shopping, taking medications, cooking food, housekeeping and doing the laundry. The summary score ranges from 0 to 14, with a score of 0 indicting totally incapable of IADL, a score of 1–5 indicating severely limited IADL, a score of 6–10 indicating limited IADL, and a score of 11–14 indicating normal IADL.

The data from the final questionnaires were statistically analysed. Based on the Cronbach’s alpha and factor analysis, the questionnaire has a high validity and reliability (Part B Cronbach’s Alpha reliability coefficient = 0.721, KMO validity coefficient = 0.739; Part C Cronbach’s Alpha reliability coefficient = 0.869, KMO validity coefficient = 0.780; Part D Cronbach’s Alpha reliability coefficient = 0.940, KMO validity coefficient = 0.935; Part E Cronbach’s Alpha reliability coefficient = 0.939, KMO validity coefficient = 0.932), indicating that this scale is reliable.

One question in Part C inquires about the floor condition in the house, and the options included a “non-slippery floor” and a “slippery floor”. However, 1,424 of the elderly participants (39.2%) responded with “without a floor”, indicating that their house floor was either earth or cement. Since the proportion selecting this option was high, we kept this as an additional optional answer.

### Statistical analyses

All data were independently entered by two investigators using EpiData 3.1. The statistical analyses were performed using SPSS version 21.0. The descriptive analyses were performed using the means (M) and standard deviations (M ± SD). The comparisons were performed using a two-tailed student’s *t*-test or single factor variance analysis (ANOVA) for the continuous variables. In all analyses, differences at P < 0.05 were considered statistically significant.

We first described the prevalence and proportion of injuries among the participants based on the injury and then compared the prevalence among groups defined by gender and age. We used the Chi-square test to determine the association between the injury status and the social demographic characteristics, chronic disease conditions, lifestyle, living environment, mental health, and ADL. Finally, a logistic regression model was performed to assess the injury risk. Ten variables were entered to study the injury patterns using a multivariate logistic regression analysis. The unintentional injuries were closely related to chronic disease, mental health and residence environment.

### Ethical considerations

The study protocol was approved by the Ethics Committee of the Tongji Medical College, Huazhong University of Science & Technology. The study was performed in accordance with the Helsinki Declaration. A letter was presented to the participants or guardian(s) explaining the aims, study procedures, and data confidentiality assurance. All participants or guardians provided a written informed consent letter before the survey was conducted.

### Limitations of the study

1. A certain degree of selection bias may exist because rural elderly participants from only one county were considered in the sampling. In a subsequent survey, more sampling points should be obtained within a province.
2. This study used a cross-sectional survey, and no interventions or follow-up analyses were performed.
